# Exogenously-Sourced Salicylic Acid Imparts Resilience towards Arsenic Stress by Modulating Photosynthesis, Antioxidant Potential and Arsenic Sequestration in *Brassica napus* Plants

**DOI:** 10.3390/antiox11102010

**Published:** 2022-10-11

**Authors:** Koser Bano, Bharty Kumar, Mohammed Nasser Alyemeni, Parvaiz Ahmad

**Affiliations:** 1 Department of Botany, Government, MVM College, Barkatullah University Bhopal (M.P.), Bhopal 462004, India; 2 Botany and Microbiology Department, King Saud University, Riyadh 11451, Saudi Arabia; 3 Department of Botany, GDC Pulwama, Jammu and Kashmir 192301, India

**Keywords:** antioxidants, cell viability, chlorophyll, metal stress, oxidative stress, sulfur-assimilation, stomatal movements, Rubisco

## Abstract

In the current study, salicylic acid (SA) assesses the physiological and biochemical responses in overcoming the potential deleterious impacts of arsenic (As) on *Brassica napus* cultivar Neelam. The toxicity caused by As significantly reduced the observed growth and photosynthetic attributes and accelerated the reactive oxygen species (ROS). Plants subjected to As stress revealed a significant (*p* ≤ 0.05) reduction in the plant growth and photosynthetic parameters, which accounts for decreased carbon (C) and sulfur (S) assimilation. Foliar spray of SA lowered the oxidative burden in terms of hydrogen peroxide (H_2_O_2_), superoxide anion (O_2_**^•−^**), and lipid peroxidation in As-affected plants. Application of SA in two levels (250 and 500 mM) protected the *Brassica napus* cultivar from As stress by enhancing the antioxidant capacity of the plant by lowering oxidative stress. Among the two doses, 500 mM SA was most effective in mitigating the adverse effects of As on the *Brassica napus* cultivar. It was found that SA application to the *Brassica napus* cultivar alleviated the stress by lowering the accumulation of As in roots and leaves due to the participation of metal chelators like phytochelatins, enhancing the S-assimilatory pathway, carbohydrate metabolism, higher cell viability in roots, activity of ribulose 1, 5-bisphosphate carboxylase (Rubisco), and proline metabolism through the active participation of γ-glutamyl kinase (GK) and proline oxidase (PROX) enzyme. The current study shows that SA has the capability to enhance the growth and productivity of *B. napus* plants cultivated in agricultural soil polluted with As and perhaps other heavy metals.

## 1. Introduction

Plants are constantly exposed to metal stress. This has become the most serious environmental constraint that affects plant growth and development [1,2,3]. Among heavy metals, arsenic (As) is a dangerous contaminant with no known biological function in plants and has higher solubility in water. It comes in diverse chemical forms, each with district levels of mobility, bioavailability, and toxicity [4,5]. Because soil structure, along with other chemical factors, determines the proportion of individual forms, soil attributes are critical for As uptake and distribution inside the plant body [1]. As can be readily absorbed by plants since it exists in two oxidation states in nature: arsenite (+3) and arsenate (+5), [4,6]. Arsenic absorption by plants is difficult to control since it is frequently mediated through transporters involved in the uptake of essential nutrients [6,7] Arsenate predominates in soil under an oxidative environment, while arsenite is prevalent under reducing conditions [8,9]. Once inside the plants, As instigates the robust production of reactive oxygen species (ROS) that results in the membrane lipid peroxidation and thus disrupts redox homeostasis of the cell [8,10]. The common ROS produced in plant cells comprises hydrogen peroxide (H_2_O_2_), hydroxyl radicals (OH), singlet oxygen (^1^O_2_), and superoxide (O_2_^•−^) anion [11]. Arsenic is one of the most dangerous metalloids for plant health and its detrimental effects disrupt metabolic and physiological responses in plants, such as plant growth [4], ROS accretion [9], and photosynthetic repercussions [9]. The minimization of As-accumulation, as well the as strengthening of the antioxidant system, have been suggested the two primary strategies for sustaining proper growth and development in plants under As stress [1,3]. Plants use and employ strategies to counter the metal/metalloid stress, including chelation (phytochelatins; PCs), osmolyte accumulation (proline), an antioxidant such as superoxide dismutase (SOD), catalase (CAT), ascorbate peroxidase (APX), glutathione reductase (GR), and non-enzymatic antioxidants, including glutathione (GSH) andascorbate (AsA) [11].

Salicylic acid (SA) is a key signaling molecule with diverse roles that regulates growth, photosynthetic, development, and biochemical mechanisms in plants [12]. The plant growth regulator modulates an array of plant reactions to abiotic stress factors, including heavy metal stress [13]. Furthermore, the positive influence of exogenously applied SA in enhancing photosynthesis and growth under As-stressed has been suggested in many plants, including *Oryza sativa* [14], *Glycine max* [15], and *Artemisia annua* [16]. SA treatment induces the synthesis of key antioxidant enzymes (SOD, APX, GR) and non-enzymatic antioxidants (AsA and GSH), all of which help to reduce cellular ROS generation and lipid peroxidation in As-stressed plants [17]. SA up-regulates the sulfur assimilation which results in the synthesis of cysteine, methionine, heavy metal chelators like non-protein thiols (NPTs), phytochelatins (PCs), metallothioneins, and various co-enzymes and vitamins. Enzymes like ATP sulfurylase (ATP-S) and O-acetylserine (thiol) lyase (OASTL) convert sulfate to Cys, which is the first product of S-assimilation. Additionally, SA also inhibits the uptake of heavy metals such as As from root to shoot via the formation of PCs and GSH [18]. Additionally, GSH is a potent S-containing metabolite that regulates cellular redox homeostasis via the AsA-GSH pathway [19].

Proline is an amino acid that plays a wide array of advantageous roles in plants, such as osmoprotectant, protein stabilization, and hydroxyl radical scavenging [20]. The accumulation of proline strengthens the resistance of plants against unfavorable environmental conditions through detoxification of ammonia and oxidative stress and by influencing the antioxidant system of the plant [21]. Many plants accumulate proline in response to heavy metal stress [22,23]. It was identified as an amicable solute for maintaining turgor pressure and protecting cellular structures [24]. After the stress is relieved, the fast breakdown of proline may supply enough reducing agents that facilitate mitochondrial oxidative phosphorylation and ATP synthesis, allowing stress recuperation and restoration of stress-induced damage [25]. In plants, glutamic acid is the precursor of proline biosynthesis. The enzymes γ –Glutamyl kinase (GK), γ-glutamyl phosphate reductase, and proline oxidase (PROX) constitute the pyrroline-5-carboxylase synthase (P5C) and regulate the proline accumulation inside the cell [26].

Plants alter their carbohydrates metabolism to cope with stressed situations, which are governed by the attainability, breakdown, and transport from source to sink [27]. Carbohydrates like sucrose, raffinose family oligosaccharides, or fructans are key free-radical quenchers that have the capability to detoxify the various type of ROS [28]. Carbohydrate metabolism is most beneficial in plant antioxidant defense systems under As stress, as recently described by Kofronova et al. [29]. Carbohydrates, in addition to their ability to quench ROS, have an indirect role in stress defense by providing energy and carbon sources for the manufacture of defense molecules, such as antioxidant enzymes [29]. Sugars like glucose modulate the harmful impacts under stressed conditions by acting as an osmolyte, maintaining the water status of the plant, controlling the pH of cytosol, and regulating membrane permeability [30]. However, in As stressed plants, inconsistent results of decrease/increased or even fluctuating sugar levels have been obtained in rice [31], wheat [32], and potato [33] plants.

*Brassica napus*, besides its economic relevance, can be employed for phytoremediation purposes because of its significantly greater biomass that accumulates more heavy metals from the soil than crop species [34]. Several studies demonstrated the ability of *B. napus* to remediate polluted soils because of its greater biomass and high heavy metal accumulation efficiency [4,35]. Enhancing the resistance of *B. napus* to metal stress is critical for efficient remediation and maintaining the growth potential of easily harvested shoots. Moreover, the physiological and biochemical basis of As stress tolerance in *B. napus* plants is still dubious and needs more answers. Based on this assumption, the current study sought to fill this gap by answering the research questions on how SA mitigates As-induced phytotoxicity in rapeseed plants and scrutinizing the various physiological and biochemical responses of the same plant in countering the As stress. The present study was also executed to explain the application of SA protecting photosynthesis and growth through increased metabolisms of S, carbohydrates, and proline. Such extensive roles of the SA in overcoming the As stress could postulate a novel approach for improving plant health in agricultural soils polluted with As.

## 2. Materials and Methods

### 2.1. Plant Material, Cultivation, and Treatments

The seeds of the *Brassica napus* cultivar, Neelam (Gobi Sarson), after sterilization, were sown in 23 cm pots that contained 4.0 kg of soil consisting of compost, sand, and peat (1:1:4). The pots carrying seeds were put in the greenhouse with a day/night temperature of 24/16 ± 33 °C, a 16/8 h light/dark cycle, and a relative humidity of 64 ± 5%.

The 200 mg As kg^−1^ soil concentration (Sodium arsenite Na_3_AsO_2_, as the source of As) was selected by studying the effects of two As concentrations (100 and 200 mg As kg^−1^ soil) studied on five *B. napus* cultivars (Neelam (Gobi Sarson), Teri Uttam Jawahar, Him Sarson, GSC-101, and NUBD 26-11) from our earlier work, Bano et al. [36]. The plants were grown with two levels of SA, i.e., 250 mM SA and 500 mM SA with or without As stress. In absolute ethanol, the SA was dissolved, then drop-by-drop combined with H_2_O (ethanol/water: 1/1000 *v/v*) and sprayed onto plant leaves using a hand sprayer at 15 DAS at concentrations of 250 and 500 mM. Only water-treated plants served as a control group.

In the experiment, each part contained four replicates (*n* = 4), and the treatments were all organized in a randomly blocked arrangement. Various morphological, photosynthetic, and biochemical variables were studied 30 days after sowing (DAS).

### 2.2. Proline Determination

The proline concentration was assessed by the ninhydrin method [37]. Leaf material (0.5 g) was standardized with 3 mL of each ninhydrin and glacial acid. The reaction mixture was extracted by using toluene. Using L-proline as a standard, the absorbance was then read at 550 nm spectrophotometrically.

γ-glutamyl kinase (GK, EC 2.7.2.11) activity and proline oxidase (PROX, EC 1.5.5.2) activity was calculated by preparing enzyme extract and by homogenizing a 550 mg leaf sample in 0.3 M TRIS-HCI buffer, pH 7.7, at 4 °C. Thereafter, the homogenate obtained was subjected to centrifugation at 40,000× *g* for 30 min. For GK and PROX tests, the pellet was assembled and utilized.

The activity of GK was measured using the Hayzer and Leisinger [38] approach, with slight modification. The frozen (−20 °C) samples were homogenized with 20 mL of 0.2 M Tris-HCI buffer carrying 1 mM 1, 4-dithiothreitol (DTT). The reaction was started by adding 0.2 mL FeCl_3_.3H_2_O and trichloroacetic acid in 5.0 M HCl after 30 min of incubation at 37 °C. The protein was then precipitated, extracted, and centrifuged at 12,000× *g* at 4 °C, and its absorbance was measured with a spectrophotometer at 540 nm.

The activity of PROX was assessed using the technique of Huang and Cavalieri [39]. The absorbance was read at 650 nm and the activity was measured in units (U) mg^−1^ protein.

### 2.3. Determination of Oxidative Stress of Plants

The lipid peroxidation was measured by calculating the quantity of thiobarbituric acid reactive substances (TBARS) in the leaves of the *Brassica napus* cultivar. Fresh tissues of the leaf (0.5 g) were ground using 0.5% 2-thiobarbituric acid in 20% trichloroacetic acid by using a pestle and mortar. The liquid was promptly chilled on an ice bath and centrifuged at 10,000× *g* for 20 min after being heated at 80 °C for 30 min. Thereafter, the absorbance of the supernatant was calculated at 550 nm by utilizing a spectrophotometer.

### 2.4. Electrolyte Leakage Determination

Electrolyte leakage was determined after washing the fresh leaves and was placed at room temperature for 24 h on a rotational shaker [40]. Then, the initial electrical conductivity (EC1) of the leaves was determined. After autoclaving the leaves for 25 min at 135 °C, the final electrical conductivity (EC2) was calculated. The following formula was employed to determine EL:

EL (%) = (EC1/EC2) × 100

The concentration of H_2_O_2_ and O_2_^•−^ was evaluated by using the protocol evaluated by Okuda et al. [41] and Wu et al. [42], and described in detail in our earlier work [36].

### 2.5. Determination of Antioxidant Process

Fresh leaves tissues (0.6 g) were homogenized using extraction buffer and 2% PVP in potassium-phosphate buffer (200 mM, pH 6.9) in a cold mortar and pestle. The supernatant acquired after centrifugation at 10,000× *g* was utilized for the evaluation of SOD (EC; 1.15.1.1) and GR (EC; 1.6.4.2) enzymes. Superoxide dismutase activity was calculated after detecting the inhibition of photochemical depletion of nitro-blue tetrazolium (NBT) [43]. Catalase activity was calculated utilizing the procedure described by Aebi [44], by observing the oxidation of H_2_O_2_ that was detected using a spectrophotometer at 240 nm. The activity of GR was assessed utilizing a slightly modified method by Foyer and Halliwell [45]. The reaction mixture included 1.0 mM oxidized GSH, 0.4 mM NADPH, and 30 mM phosphate buffer (pH 8.2). For the evaluation of APX (EC; 1.11.1.11), 3.0 mM ascorbate was supplemented with extraction buffer. The activity of APX was determined using the technique evaluated by Nakano and Asada [46], which involves observing a decrease in ascorbate at 290 nm. The details are provided in our earlier work [36].

The enzyme recycling technique of Griffith [47] was utilized to measure reduced glutathione (GSH) spectrophotometrically at 425 nm. For this, 6% sulphosalicylic acid was used to homogenize fresh leaves (500 mg) and then 12,000× *g* for 25 min. The reading of absorbance was measured at 416 nm after a 2 min incubation period.

### 2.6. Determination of Photosynthetic Characteristics and Growth Characteristics

The gas exchange indices were calculated with the aid of an infrared gas analyzer (IRGA; LiCOR-7680, Portable photosynthesis system, Bio-science, Washington, DC, USA). Attributes such as net photosynthesis (P_N_, stomatal conductance (gs), and intercellular CO_2_ concentration (C_i_) were calculated on a sunny day between 10:00 and 11:00 h at photosynthetic active radiation (PAR) above 740 µmol m^−2^ S^−1^ and 445 ± 4 µmol^−1^ atmospheric CO_2_ concentrations.

A quantity of chlorophyll in the uppermost, fully expanded leaves was measured utilizing a SPAD chlorophyll meter (620-DL PLUS, Spectrum Technologies, Aurora, IL, USA).

The activity of Rubisco was measured by using the technique of Usuda [48], which involves monitoring NADH oxidation at 340 nm at 30 °C. The details are provided in our earlier publication [36].

For plant growth attributes, the uppermost leaves were dried in an oven at 85 °C. The leaf area was measured from the remaining leaves of the plant using a leaf area meter (LA 25, Systronics, New Delhi, India).

### 2.7. Determination of Arsenic and Sulfur Content

Using an atomic absorption spectrophotometer, the concentration of As was determined. The samples of roots and leaves were dried for about 2 days at 75 °C in an oven. The dried samples from leaf and roots in powdered form were subjected to a solution of concentrated HNO_3_:KClO_4_ in the ratio of 3:1 (*v/v*). The observations were recorded using atomic absorption spectrophotometry.

The leaf S content was determined by the turbidimetric method in an oven-dried leaf (0.4 g) in the powdered form [49]. The leaves were then subjected to a solution containing nitric acid and perchloric in a volumetric ratio of 85:15. The recordings were then measured spectrophotometrically at 430 nm after turbidity started developing.

### 2.8. Determination of Non-Protein Thiols (NPTs) and Total Phytochelatins (PCs)

The level of foliar non-protein thiols (NTPs) and total phytochelatins (PCs) was accessed by using the method of Lou et al. [50], with slight modifications. Fresh leaf samples (2.0 g) were homogenized with 10% sulfosalicylic acid. The resultant solution was then centrifuged at 12,000× *g* for 25 min. After a 40 min incubation period, the absorbance of the reaction mixture was measured on a spectrophotometer at 425 nm. The PCs were calculated as total NPTS minus GSH.

### 2.9. Determination of Carbohydrates

The concentration of total soluble sugar (TSS) was calculated using anthrone as a reagent [51]. In 5 mL of 95 percent (*v/v*) ethanol, fresh leaves and roots (500 mg) were homogenized individually. The insoluble fraction of the extract retained the determination of starch content. The total soluble sugar (TSS) concentrations were determined by mixing the extract with produced anthrone reagent and recording absorbance at 630 nm using glucose as standard.

Starch content was determined by carrying out the acid hydrolysis of the leaf material [52]. Next, 55% perchloric acid was added to the extract and left for incubation for 30 min. To this mixture, 20 mL of cold anthrone reagent was added and then heated at 90 °C followed by cooling in an ice bath. The absorbance was measured spectrophotometrically at 635 nm, and starch concentration was calculated using a starch standard curve.

Following the method of Krishnaveni, et al. [53], glucose contents were calculated in fresh leaves and roots (100 mg) individually using a glucose oxidase peroxidase reagent. 1 mL of glucose oxidase peroxidase reagent was added to 0.25 mL of deproteinized extracts after diluting them with an equivalent volume of distilled water. The reaction process was terminated by adding 1 mL of 3 N-HCl to the reaction mixture and measuring the absorbance spectrophotometrically at 550 nm.

Using the technique of Jones et al. [54], sucrose content in fresh leaves and roots (1 g) was tested. The reaction mixture consisted of 200 mM imidazole buffer (pH 7.6), 80 mM imidazole base, and 1 mM adenosine triphosphate (APT). Thereafter, the absorbance was spectrophotometrically determined at 340 nm UV. Each reaction mixture received 85 microliters of glucose-6-dehydrogenase (70 units mL^−1^), which was thoroughly mixed and re-read after about 5 min. Parallel blanks were run and sucrose content was determined from a standard curve of sucrose.

### 2.10. Studying Root Cell Viability with Confocal Laser Microscopy

To visualize the cell death as red fluorescence, clean and thin slices of roots were immersed in a 50 µM propidium iodide (PI) solution. Root samples were studied using a confocal microscope (Olympus Fluoview TM-FV1000, Olympus Life Sciences, Tokyo, Japan) after being thoroughly washed at excitation of 420–485 nm, emission ≥515 nm, and processed using Fluoview FV10 software, ver 1.7 (Olympus Life Sciences, Tokyo, Japan). The studies were done on 30 day old roots.

### 2.11. Physiological Measurement of Guard Cells

Fresh leaves were immersed in 4% glutaraldehyde for fixation and then rinsed with distilled water. Next, the leaves were dehydrated using ethanol series (50 to 100%). Ultimately, the material was dehydrated with liquid CO_2_ after being transferred to pure isoamyl acetate for 1 h. The gold-palladium-coated dehydrated specimen were visualized under the microscope. Analyses were carried out on leaves that were 30 days old. The images were captured using Carl Zeiss EVO 40 scanning electron microscope (Zeiss, Aalen, Germany).

### 2.12. Statistical Analysis

Data were analyzed statistically by analysis of variance (ANOVA) using SPSS v18.0 for Windows (IBM Corporation, New York, NY, USA). The least significant difference (LSD) was calculated for mean separation for significant differences among treatments at *p <* 0.05 levels.

## 3. Results

### 3.1. Impact of Salicylic Acid on Growth Metrics during As Stress

**Figure 1:**
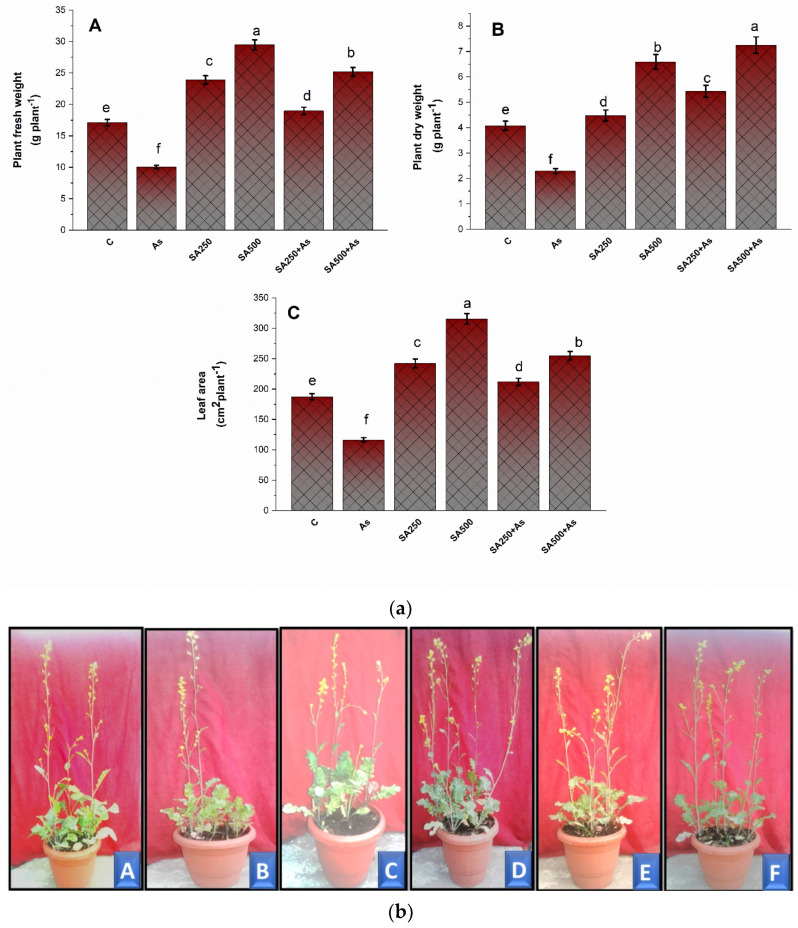
(**a**) Plant fresh weight (**A**), dry weight (**B**), and leaf area (**C**) in As stress-tolerant *Brassica napus* cv. Neelam at 30 days after growth. Plants supplied As (200 mg As kg^−1^ soil) were exposed with foliar applied SA (250 and 500 mM). The data are represented as treatments mean ± SE (*n* = 4). The LSD test represents the data followed by the same letter are not considerably different by Duncan at *p <* 0.05. C, control; As, arsenic; SA, salicylic acid. **(b)** Plant phenotype under different treatments of SA with or without As stress. The various treatments include (**A**) control, (**B**) 200 mg As kg^−1^ soil, (**C**) 250 mM SA, (**D**) 500 mM SA, (**E**) 250 mM SA + As, and (**F**) 500 mM SA + As SA, salicylic acid; As, arsenic.

Arsenic has a detrimental impact on plant health and contributes to toxicity by causing oxidative stress through the generation of ROS. Exposed *B. napus* plants showed a significant (*p* ≥ 0.05) drop in growth parameters such as fresh weights by 41.2%, dry weight by 44.7%, and leaf area by 37.2% in comparison to control (Figure 1(aA–aC)). To minimize the harmful effect of As, we used two doses of salicylic acid (250 mM SA and 500 mM SA) as a foliar spray. With the application of 250 and 500 mM SA, there was a significant increase in the growth parameters like FW by 12.5% and 41.3%, DW by 33.9%, and 77.5%, and LA by 14.9% and 36.2%, respectively, as compared to the control plants (Figure 1(aA–aC)). The results were verified using plant phenotype and each treatment reflected their concurrent phenotype (Figure 1(bA–bE)). The plants exposed to 200 As showed stunted growth when grown in the pots of same size and soil as other plants (Figure 1(bB)). As can be seen in Figure 1(bE,bF), plants treated with 250 and 500 mM SA with As clearly reversed the deleterious impacts of As on plant growth.

### 3.2. Impact of SA on Photosynthesis Parameters under As Stress

**Figure 2:**
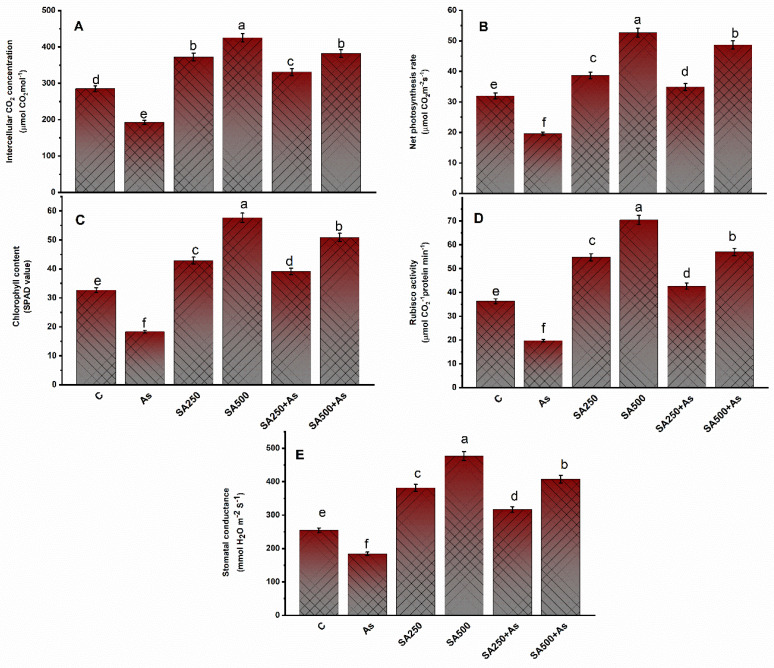
Intercellular CO_2_ concentration (**A**), Net photosynthesis (**B**), Chlorophyll content (SPAD values) (**C**), Rubisco activity (**D**), and Stomatal conductance (**E**) in As stress-tolerant leaves of *Brassica napus* cv. Neelam at 30 days after growth. Plants supplied As (200 mg As kg^−1^ soil) were exposed with foliar applied SA (250 and 500 mM). Data are shown as mean ± SE (*n* = 4). The LSD test represents the data followed by the same letters are not considerably different by Duncan at *p* < 0.05. As, arsenic; Rubisco, ribulose-1, 5-bisphosphate carboxylase/oxygenase; SA, salicylic acid.

Arsenic stress considerably (*p* ≥ 0.05) decreased the photosynthetic components of plants such as net photosynthesis (P_N_) by 38.4%, stomatal conductance (gs) by 27.8%, intercellular CO_2_ content (Ci) by 33.7%, Rubisco activity by 45.6%, and chlorophyll content by 42.1% in contrast to control (Figure 2A–E). Foliar spray of SA significantly raised all the studied photosynthesis characteristics under no-stress conditions. Further, supplementation of the As stressed plants reduced the detrimental effect of As on photosynthesis parameters, with the 500 mM SA proving to be the best dose and more effective than 250 mM SA. The exogenous application of 250 mM SA and 500 mM SA increased the photosynthetic attributes such as P_N_ by 27.8% and 52.4%, Ci by 15.6% and 33.8%, chlorophyll content by 19.2% and 55.5% Rubisco activity by 17.9% and 56.3%, and gs by 24.4% and 60.7%, compared to control plants (Figure 2A–E).

### 3.3. SA Reduces Oxidative Stress in Brassica napus Plants under As Stress

**Figure 3:**
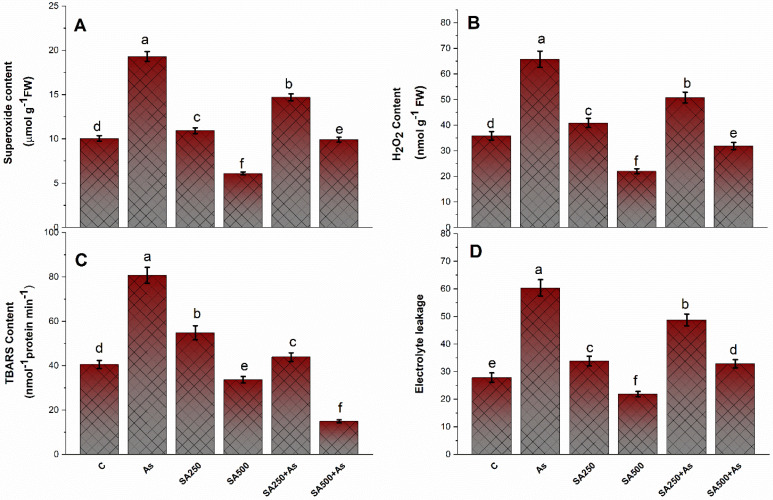
Superoxide content (**A**), H_2_O_2_ content (**B**), TBARS content (**C**), and Electrolyte leakage (**D**) in *Brassica napus* cultivar Neelam at 30 days after growth. Plants supplied As (200 mg kg^−1^ soil) were exposed with foliar applied SA (250 and 500 mM). Data are presented as mean ± SE (*n* = 4). The LSD test represents the data followed by the same letters, which are not considerably different to those by Duncan at *p* < 0.05. TBARS, thiobarbituric acid reactive species; H_2_O_2_, hydrogen peroxide; SA, salicylic acid.

Plants treated with As had great levels of oxidative stress, which leads to the formation of ROS in plants. The greater As content increased the H_2_O_2_ content by 83.1%, O_2_^•−^ content by 91.5%, TBARS content by 94.2%, and electrolyte leakage (EL) by 83.2% in comparison to control plants (Figure 3A–D). SA application in two doses markedly and significantly reduced the H_2_O_2_ content by 22.6% and 51.5%, O_2_^•−^ content by 24.7% and 48.1%, TBARS content by 45.3% and 81.4%, and EL by 19.7% and 45.9%, respectively, by using 250 and 500 mM, respectively, a contrast to the control plants (Figure 3A–D).

### 3.4. SA Up-Regulates the Antioxidant Metabolisms in B. napus under As Stress

**Figure 4:**
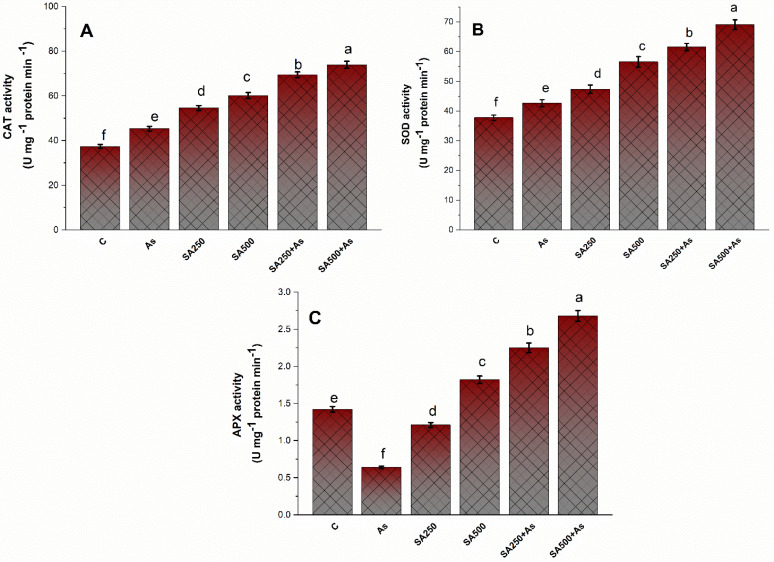
CAT activity (**A**), SOD activity (**B**), APX activity (**C**) in *Brassica napus* cultivar Neelam at 30 days after growth. Plants supplied As (200 mg kg^−1^ soil) were exposed to SA (250 and 500 mM). Data are shown as mean ± SE (*n* = 4). The LSD test represents the data followed by the same letter are not considerably different by Duncan at *p* < 0.05, As, arsenic; APX, ascorbate; CAT, catalase; SOD, superoxide dismutase; SA, salicylic acid.

Application of As to of *B. napus* cultivar elicited a slight but significant enhancement to the enzymatic activities of CAT, SOD, and APX by 16.6%, 54.3%, and 21.5%, respectively, in contrast, to control plants (Figure 4A–C). The enzyme activities were considerably greater in stressed plants compared to unstressed plants. On application of 250 mM SA, CAT activity increased by 85.1%, the activity of SOD increased by 63.4%, and activity of APX increased by 58.5%. Similarly, with the application of 500 mM SA, the activity of CAT, SOD, and APX increased by 86.9%, 88.2%, and 97.1%, respectively, in contrast to control plants (Figure 4A–C).

### 3.5. Impact of Salicylic Acid on the Accumulation of Proline under As-Stress

**Figure 5:**
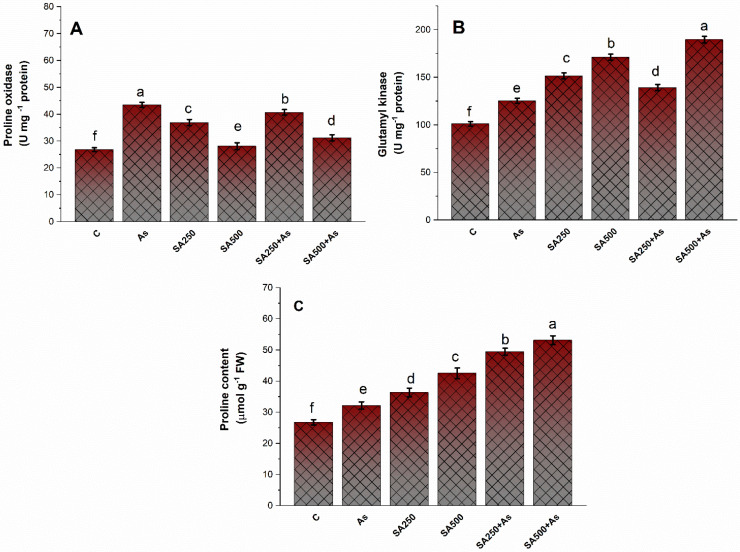
Proline oxidase (**A**), Glutamyl kinase (**B**), and Proline content (**C**) in As stress-tolerant *Brassica napus* cultivar Neelam at 30 days after growth. Plants supplied As (200 mg As kg^−1^ soil) were exposed to SA (250 and 500 mM). Data are shown as mean ± SE *(n* = 4). The LSD test represents the data followed by the same letter are considerably different by Duncan at *p* < 0.05. As, arsenic; DAS, days after sowing; SA, salicylic acid.

Proline metabolism caused the reduction of the detrimental impact of As toxicity on the plants under stress conditions. There was a slight increase in proline content (20.2%), γ-glutamyl kinase (GK) activity (36.1%), and proline oxidase (PROX) activity (23.4%) in As-exposed plants (Figure 5A–C). This increase was further boosted with the exogenous application of SA and a differential increase was detected at both levels. Particularly, foliar spray of 500 mM SA enhanced proline content by 98.2% GK activity by 87.2% and PROX activity by 91.1%, when compared to the control group (Figure 5A–C).

### 3.6. Impact of Salicylic Acid on S-Assimilation during As Stress

**Figure 6:**
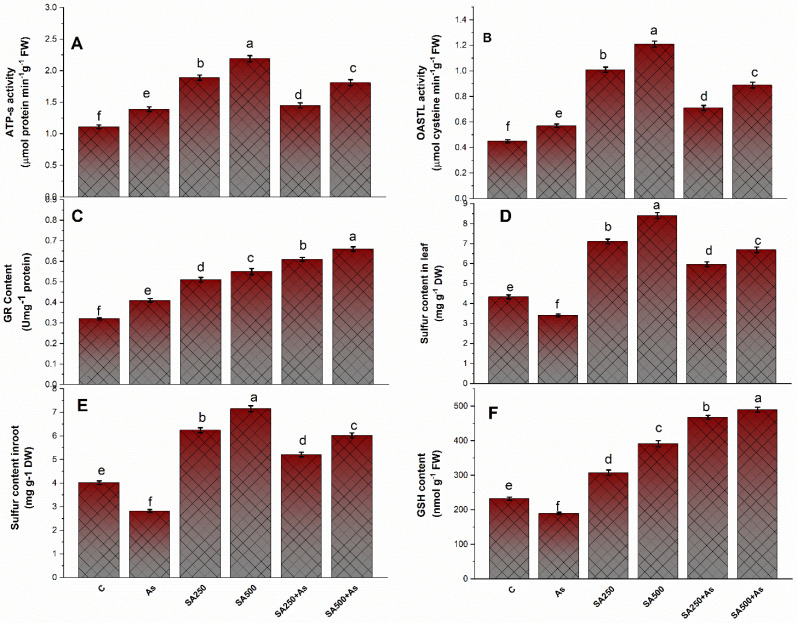
ATP-S activity (**A**), OASTL activity (**B**), GR content (**C**), Leaf sulfur content (**D**), Root sulfur content (**E**), and GSH content (**F**) in As stress-tolerant *Brassica napus* cv. Neelam at 30 days after growth. Plants supplied As (200 mg kg^−1^ soil) were exposed to SA (250 and 500 mM). Data are shown as mean ± SE (*n* = 4). The LSD test represents the data followed by the same letter are not considerably different by Duncan *p* < 0.05. OASTL, O-acetyserine (thiol) lyase; NPTs, non-protein thiols; PCs, phytochelatins; ATP-S, ATP sulfurylase; SA, salicylic; As, arsenic.

Treatment of As declined the activities of ATP-S, OAS-TL, and GR by 25.6%, 27.2%, and 28.5% in contrast to control plants (Figure 6A–C). Both concentrations of SA boosted the activity of the above-mentioned enzymes during stressed and un-stressed conditions. However, the plants treated with 500 mM SA in the presence of As demonstrated a considerable increase in ATP-S, OAS-TL, and GR activity by 64.4%, 97.2%, and 96.2% when compared to the control plants (Figure 6A–C).

Under the influence of As, the GSH content and S content in roots and leaves of *B. napus* decreased by 18.6%, 29.1%, and 21.9%, respectively, when compared to control (Figure 6D–F). The GSH levels decreased significantly by 18.4% under As exposure, but the leaf Cys content increased by 39.8% when compared to control plants. It is enhanced even more when SA (250 and 500 mM) is applied in two doses. The concentration of S and GSH enhanced with the exogenous application of SA (250 and 500 mM), and the highest increase was observed in plants treated with 500 mM SA (Figure 6D–F). Furthermore, the supplementation of 500 mM SA reduced the adverse effects of As more prominently than 250 mM SA. The 500 mM SA raised the sulfur content in roots and leaves by 49.8% and 54.2%, respectively, whereas the GSH content increased by 2.11 folds under As stress compared to the respective control (Figure 6D–F).

**Figure 7:**
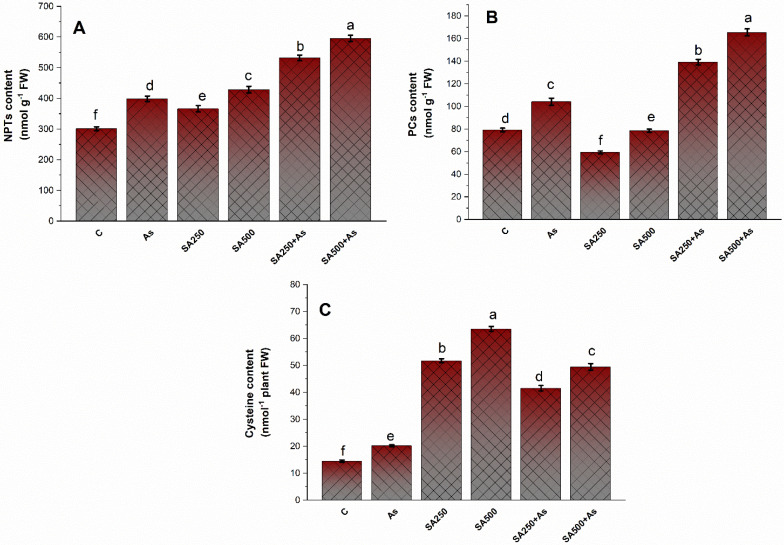
NPTs content (**A**), PCs content (**B**), and cysteine content (**C**) in As stress-tolerant *Brassica napus* cv. Neelam at 30 days after growth. Plants supplied As (200 mg As kg^−1^ soil) were exposed to SA (250 and 500 mM). Data are shown as mean ± SE (*n* = 4). The test represents the data followed by the same letter are not considerably different by Duncan at *p* < 0.05. As, arsenic; GSH, Glutathione; GR, glutathione reductase; SA, salicylic.

The NPTs, PCs, and Cys levels were calculated in leaves of *B. napus* cultivar Neelam in both As stressed and un-stressed conditions (Figure 7A–C). The NPTs, PCs, and Cys content were higher (32.7% 31.2%, and 26.8%, respectively) in As stressed plants. Plants treated with 250 and 500 mM SA increased the accumulation of NPTs by 75.7% and 97.4% PCs content by 76.4% and 98.2%, and Cys content by 56.5% and 69.1%, respectively, when compared to control plants (Figure 7A–C).

### 3.7. Impact of SA on the Carbohydrate’s Metabolism under As Stress

**Figure 8:**
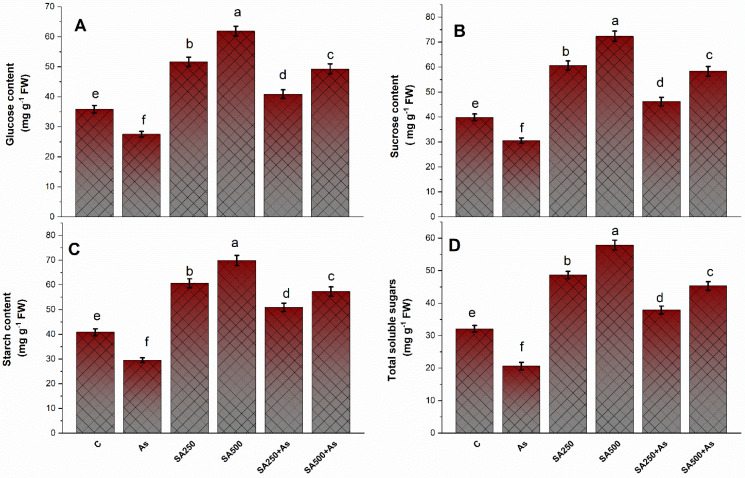
Glucose content (**A**), Sucrose content (**B**), Starch content (**C**), and Total soluble sugars content (**D**) in *Brassica napus* cv. Neelam at 30 days after growth. Plants supplied As (200 mg As kg^−1^ soil) were exposed to SA (250 and 500 mM). Data are shown as mean ± SE (*n* = 4). The LSD test represents the data followed by the same letter are not considerably distinct by Duncan at *p* < 0.05. As, arsenic; SA, salicylic acid.

The content of carbohydrates such as glucose, sucrose, total soluble sugars, and starch content in leaves of *B. napus* was significantly (*p* ≤ 0.05) decreased by 18.9%, 21.5%, 29.8%, and 27.7% under As stress as compared to control (Figure 8A–D). In non-stressed plants, treatment with both levels of SA considerably boosted the content of glucose, sucrose, total soluble sugars, and starch, and both the levels of SA were extremely effective. More effect was perceived with 500 mM SA. Further, in As-affected plants, there was an obvious increase in the content of glucose, sucrose, total soluble sugars, and starch, which reversed the inhibitory impact of As on carbohydrate metabolism. Foliar feeding with 500 mM SA enhanced glucose, sucrose, total soluble sugars, and starch content by 41.2%, 46.9%, 49.5%, and 40.5% in contrast to the control (Figure 8A–D).

### 3.8. Impact of SA on Arsenic Accumulation in Roots and Leaves

**Figure 9:**
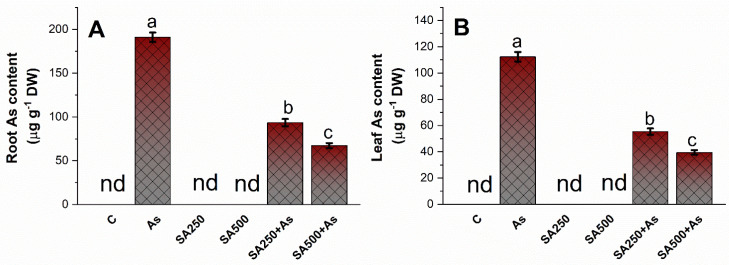
Root As content (**A**) and Leaf As content (**B**) in As stress-tolerant *Brassica napus* cv. Neelam at 30 days after growth. Plants supplied As (200 mg kg^−1^ soil) were exposed to SA (250 and 500 mM). Data are shown as mean ± SE (*n* = 4). The LSD test represents t data followed by the same letter are not considerably different to Duncan at *p* < 0.05. As, arsenic; nd, not detected; SA, salicylic acid.

The *B. napus* cultivar treated with As observed an increase in As accumulation in roots and leaves (Figure 9A,B). The amount of As accumulated in roots (195.11 µg g^−1^ DW) was higher than in leaves (131.41 µg g^−1^ DW). SA at both concentrations significantly (*p* ≤ 0.05) reduced the As content in the roots and leaves of the plants. The reduction in root As content with 250 mM SA was 51.2%, and with 500 mM SA it was 63.6%, respectively, compared to As stressed plants. Similarly, in leaves, 250 mM SA showed a reduction of 54.4% of As content while 500 mM SA lowered As concentration by 65.7%, compared to only As treated plants (Figure 9A,B).

### 3.9. Effect of SA on Cell Viability and Stomatal Studies under As-Stress

**Figure 10:**
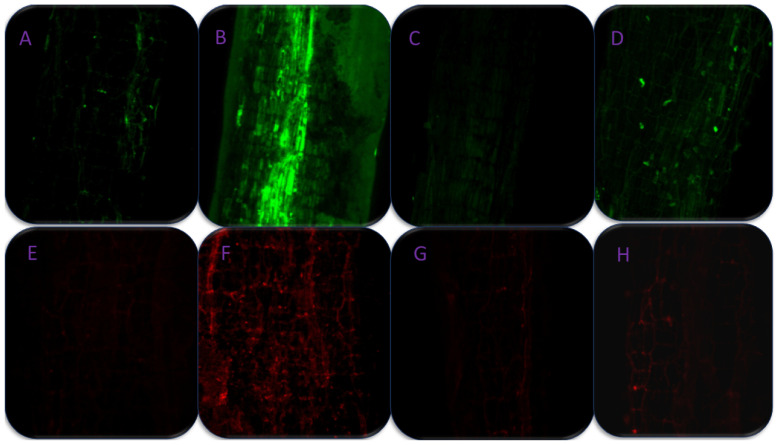
Confocal microscopic images of H_2_O_2_ production in roots using H_2_DCFDA staining (**A**–**D**) and cell viability test (**E**–**H**) by propidium iodide staining in 30 day old roots and leaves, respectively, of *Brassica napus* cultivar Neelam under control (**A**,**E**), 200 mg As kg^−1^ soil (**B**,**F**), 250 mM salicylic acid with As (**C**,**G**), and 500 mM salicylic acid with As (**D**,**H**). H_2_DCFDA, 2′,7′-dichlorodihydrofluorescein diacetate; As, arsenic.

As-induced H_2_O_2_ accretion was seen in roots of *B. napus* stained with H_2_DCFDA, a marker for ROS primarily H_2_O_2_ in cells (Figure 10A–D). The H_2_DCFDA enters passively inside the cells and is oxidized with H_2_O_2_. The non-fluorescent H_2_DCFDA transforms into highly fluorescent 2′,7-dichlorofluorescein (DCF). From the results, it is clear that the plants exposed to As showed a higher intensity of green fluorescence (Figure 10B).

The foliar spray of 250 and 500 mM SA to As stressed plants minimized the H_2_O_2_ content, as evident by lower green fluorescence, and showed a similar response to control plants (Figure 10C,D).

Propidium iodide (PI) is a staining dye used to visualize dead cells as red fluorescent spots. Stressed plants’ root cells were less viable and displayed more stains (Figure 10F). On the other hand, SA application at both levels reversed As-induced cell death, and SA-treated plants responded similarly to control plants (Figure 10E,G,H).

**Figure 11:**
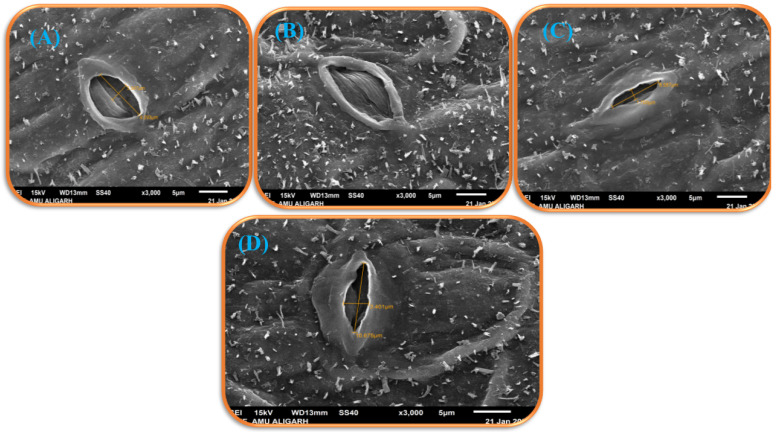
Stomatal response under a scanning electron microscope at 3000 × in 30 days old leaves of As stress-tolerant *Brassica napus* cv. Neelam under control (**A**), 200 mg As kg^−1^ soil (**B**), 250 mM salicylic acid with As (**C**), and 500 mM salicylic acid with As (**D**). As, arsenic.

Electron microscopy was utilized to study the stomatal behavior in response to As stress and SA supply (Figure 11A–D). In As-stressed plants, the stomatal aperture changed significantly and appeared partially closed with deformed guard cells (Figure 11B). However, the stomata in leaf samples of control, 250 mM SA+ As, and 500 mM SA + As were normal, with wide and open stomatal aperture (Figure 11A,C,D).

## 4. Discussion

Plant growth regulators influence the tolerance mechanism operations against metal stress in plants through regulating several physiological processes and biochemical pathways [55]. Salicylic acid is a multi-faced signaling molecule that protects plants against metal stress tolerance [56,57]. In our research, the exogenous application of SA enhanced plant growth, photosynthesis, and biochemical parameters that were impaired as a consequence of As phytotoxicity. Arsenic toxicity is detrimental to abiotic factors affecting plant growth [1], photosynthesis [58], and crop yield [4]. It also has severe effects on humans and is considered a potential carcinogenic agent [2,59]. In the current study, we explained the tolerance mechanism of *B. napus* plants under As stress through the supplement of SA in a dose-dependent manner. The exogenous treatment of SA (250 mM SA and 500 mM SA) enhances growth, photosynthesis, and antioxidant mechanisms, and increases the proline and carbohydrate content in mitigating the As-induced toxicity. It was observed that of two levels of SA employed, 500 mM SA was more efficacious in diminishing the toxic consequences of As and enhancing the highest growth and photosynthesis of the plants.

**Salicylic acid enhances plant growth by decreasing the As toxicity**

The exposure of plants to As decreased the plant growth parameters like plant fresh weight, dry weight of the plant, and leaf area, and increased the As accumulation within plant tissues (Figure 1A–C). Arsenic, after the absorption by the roots, can severely stifle plant growth and biomass accumulation by lowering the chlorophyll concentration, resulting in a reduction in photosynthetic rate [60]. The reduced growth of the plants, because of the As stress, arises predominantly due to the extensive ROS formation, inhibition of photosynthesis, and metabolic enzymes [4,9]. Furthermore, the requirements for high energy and biomolecule synthesis in providing As tolerance may also result in a decline in growth in the plants under higher As concentrations. Exogenous SA application ameliorated the toxic effects caused by As through cell membrane stabilization, change of hormonal balance, ion inactivation, and stimulation of antioxidants [15,61]. The restoration of plant growth by SA might be auxin-mediated or related to the reduction in As accumulation in the shoots of the plants [18,62]. SA causes the activation of auxin synthesizing transcriptional factors responsible for the production of auxin [62]. Foliar application of SA reduces the translocation of heavy metals from roots to shoots by acting as a metal-binding site that prevents metals from being translocated from roots to shoots and the apoplast [63]. Moreover, our study reported the higher accumulation of metal chelators like NPTs and PCs that resulted in the subsequent reduction of As in plant tissues. Lower accumulation of As in plant parts could also be a reason for higher growth. Therefore, the optimal supplementation of SA can increase plant growth under stress conditions and minimize As effects on growth inhibition.

**Salicylic acid reverses the As-induced photosynthetic inhibition and improves photosynthetic capacity**

Arsenic disrupts the process of photosynthesis by interacting with enzymes, interfering with the production of photosynthetic pigments or accelerating their degradation [4]. In our results, it was observed that As-treated plants revealed a remarkable decrease in photosynthesis attributes such as chlorophyll content, leaf gas exchange variables, and Rubisco activity, thus lowering the photosynthetic capacity of the plant (Figure 2A–E). Exogenous application of 250 and 500 mM SA to *B. napus* plants resulted in the mitigation of As-induced effects on observed photosynthetic parameters (Figure 2A–E). This increase in photosynthetic capacity, chlorophyll content, and activity of the Rubisco enzyme could be attributed to the allocation of C, S, and N for the biosynthesis of pigments, enzymes, and proteins [64]. Plants treated with SA with high photosynthetic NUE and –SUE had stronger potential to allocate N and S to the leaf by increasing the activity of the ATP-S enzyme [65]. Under As stress, SA application boosted photosynthetic parameters such as chlorophyll content, stomatal conductance, and Rubisco activity more efficiently, as discussed earlier in *wheat* [66] and *Artemisia annua* [67]. The As-mediated decrease in the photosynthetic variables in the *B. napus* cultivar might also be due to the closing of stomata, which limits the availability of internal CO_2_. Photosynthetic rate rises in As-treated plants with exogenous SA were presumably due to the Rubisco enzyme being highly allocated by nitrogen (N) and its effect on stomata, which increased stomatal conductance [65,66]. The deficiency of salicylic acid decreased the level of chlorophyll content, inhibited the Rubisco expressing genes, and also decreased the catalytic activity of Rubisco, which causes alterations in the electron transport chain, in addition to loss of grana, which can result in photorespiration [65]. It has been established that as a result of As, stress demand for SA increased for the proper functioning of the photosynthetic machinery, antioxidant defense mechanism, and balancing of protein synthesis for photosynthesis [65]. In this investigation, scanning electron microscopy images demonstrate that SA-treated plants had wider stomatal openings. SA regulates stomatal opening through the up-regulation of various signal transduction pathways (Figure 11A–C).

**Salicylic acid improves Proline accumulation under As stress.**

Proline is a vital amino acid that plays a key role in the plant protection system against heavy metal stress [23]. Plants accumulate a large quantity of proline when exhibiting heavy metal stress [23,24]. Proline reduces heavy metal stress by acting as an osmoprotectant, a ROS quencher, and a heavy metal chelator, which helps to alleviate heavy metal stress [68]. In this study, we observed that proline concentration raised considerably in the leaves of the Neelam cultivar of *B. napus* plants under As stress (Figure 5A–C). Exogenously supplied SA increased the synthesis of proline by increasing the activity of proline synthesizing enzymes under As-stress circumstances (Figure 5A–C). Proline is an indirect ROS scavenger because it causes the synthesis of NADP^+^, which results in the formation of proline, by the utilization of NADPH for the reduction of glutamate to Pyrroline-5-Carboxylate (P5C), and then from Pyrroline-5-Carboxylate to proline [69]. The NADP^+^ is further subsequently employed as an electron acceptor, preventing the formation of singlet oxygen and reactive oxygen species (ROS) under stressful circumstances [69]. Proline plays a diverse role in maintaining cellular water status, membrane stabilization, ROS scavenging, and as an enzyme chaperone at the time of stress [20,23]. Proline has also been documented to work as a molecular chaperone, protecting protein integrity and modulating the activities of several key enzymes [70]. The exogenous application of SA exhibits an increase in the activity of both the γ-glutamyl kinase (γ-GK) and glutamic-γ-semialdehyde dehydrogenase (GSA-GK) which play a key role in the synthesis of proline. SA treatment increased the synthesis of proline under As stress. Our results are in agreement with those of Nazar et al. [70], who described that exogenous supplementation of SA enhanced the proline concentration under the As stress condition.

**Salicylic acid enhances carbohydrate metabolism under As stress**

The carbohydrate synthesis, translocation, storage, and utilization enhanced the yield and productivity of the crops [71]. Total carbohydrates are the main source of energy for plants and are also a key factor in stress-related growth and development. Carbohydrates can effectively quench free radicals, protecting against oxidative stress [27]. Carbohydrates, especially glucose, sucrose, raffinose family oligosaccharides, and fructose, act as signaling molecules and take part in ROS detoxification under abiotic stresses [29]. Furthermore, soluble sugars and starch play a crucial role in signal transduction, membrane lipid production, osmotic control, carbon, and energy supply. [29]. In our results, the total structural carbohydrates (TSC) such as glucose, fructose, and sucrose were accumulated in the leaves of *B. napus* cultivars as a defensive response from heavy metal stress, induced by As. As can bind to thiol group of enzymes and drastically influence their function. The reduction of starch content under As stress was mainly due to the inhibition of starch synthase activity and degradation of enzymes such as starch phosphorylase and β-amylase [31]. The carbohydrate synthesis hampered the Rubisco activity and photosynthesis in *B. napus* (Figure 8A–D). In this study, exogenous application of SA (250 and 500 mM SA) remarkably decreased the increase in As-induced total carbohydrate content (Figure 8A–D). This indicates that SA increased the formation of total carbohydrates under As stress by modulating the metabolism of starch and sugar in the leaves of *B. napus* plants. SA treatments promoted the activity of enzymes (Sucrose phosphate synthase, sucrose synthase, and acid invertase) involved in sugar production [72,73]. In our study, in *B. napus* cultivars Neelam (Gobi Sarson), the arsenic treatment affects glucose metabolism in both leaves and roots. This is following the results on relative As-sensitive tomato previously described by Jha et al. [31] and Gupta and Seth [74]. The activities of starch-degrading enzymes (α-amylase and β-amylase) show that starch in the leaves rapidly hydrolyzed to accessible glucose under stress conditions [31]. Our results are parallel with the proteomic studies of Garg [75], which revealed that exogenous supplementation of SA can up- or down-regulate certain proteins and carbohydrate metabolism enzymes in stressed plants [75].

**Salicylic acid reduced the oxidative stress under As stress.**

The accumulation of As in tissue is the chief contributor to As stress in plants. Arsenic stress gives rise to the generation of oxidative stress mostly by inhibiting electron transport during the photosynthetic process [1,76]. An increase in the TBARS (a lipid peroxidation marker), H_2_O_2_ content, and O_2_^−^ are common indicators of oxidative damage. Furthermore, As stress exacerbated electrolyte leakage due to greater O_2_^•−^, TBARS, and H_2_O_2_ levels, as previously noted in soybean by Chandrakar et al. [2]. These biomolecules disrupt the ion exchange capability of the cell membrane and thus impact metabolic processes linked to cell membrane functioning [77]. In the present study, the treatment of SA in a dose-dependent manner in the presence of As restricted As uptake and accumulation. Exogenous SA treatment remarkably diminished the content of As in leaves and roots, with the drop in leaves being greater than in roots. Our results are concurrent with what has been observed in rice [14]. In addition to this, our findings are consistent with prior research that has demonstrated that SA can prevent the excessive accumulation and transport of metals or metalloids to plants including As in rice [14] and Cd in both *wheat* and *soybean* [78,79]. The reduction in As accumulation and oxidative stress under stress conditions could be attributed to the SA-induced synthesis of metal chelators like NPTs and PCs and antioxidants, which also protected the *B. napus* cultivar Neelam from As effects (Figure 7A–C). These findings are in agreement with Kaya et al. [12], Nazar et al. [65], and Hasanuzzaman et al. [80]. This could also be owing to SA-induced down-regulation of root to shoot As transporters [56]. The requirement for more thiols for the elimination of excess As and ROS in plant tissues was fulfilled by enhanced S-assimilation via increased activity of ATP-S and OAS-TL, consequently resulting in effective ROS detoxification (Figure 6A–C). In the present investigation, the application of SA at 500 mM concentration maximally reduced the cell death in root cells of *B. napus* plants. The reduction could be due to the presence of SA in roots that enhanced the antioxidant formation in roots. Saidi et al. [61] discovered that an increased antioxidant enzyme reduces ROS reaction with nuclear DNA, which might explain the reduction in DNA damage. These findings indicate that SA modulates plant growth and development by maintaining the ratio of anti- and pro-oxidants in cells.

**Salicylic acid enhanced antioxidant metabolism and maintains ROS homeostasis.**

The mitigative effect of SA on oxidative stress is due to the mere participation of thiols and multiple pathways to control excessive ROS inside plants [81]. Antioxidants (both enzymatic and non-enzymatic) efficiently detoxify the excessive ROS inside the cell. SA is considered to enhance the antioxidant defense mechanisms, which could increase plant growth under metal-stressed environments [79]. In our research, the decrease in ROS accumulation was accompanied by a rise in antioxidant enzyme activity such as CAT, APX, and SOD (Figure 4A–C). The greatest rise in both enzymatic antioxidants (CAT, APX, SOD, and GR) and non-enzymatic antioxidants (GSH) was with 500 mM SA (Figure 4A–C and Figure 6C,F). An earlier study found that the treatment of SA increased the activity of antioxidants (SOD, CAT, APX, GR, and GSH), which caused the reduction of ROS accumulation in plants that were exposed to heavy metals [18]. The GSH operates via an AsA-GSH cycle, which is a key mechanism for the detoxification and quenching of excessive ROS and maintaining proper cellular redox balance inside the cell under stress [82].

Additionally, GSH is the most abundant soluble antioxidant inside cells, which launches the antioxidant defense system so that they can adjust to environmental challenges and stresses [12,82]. Accordingly, GSH can efficaciously scavenge ROS and participate in the formation of phytochelatins (PCs), which bring about the vacuolar sequestration of metals/metalloids in plants [11,82]. The antioxidant GSH also helps in the protection of cell membranes from peroxidation [11,82]. Thus, the 500 mM SA application perceptibly participates in the reduction of oxidative stress by involving antioxidants that result in the protection of *B. napus* plants under As stress.

**Role of salicylic acid-induced S-assimilation in As stress tolerance**

Exogenous application of SA successfully up-regulated the uptake and assimilation of sulfate in *Brassica napus* plants under As stress [65]. In the current study, the exogenous supplementation of SA raised the activities of S-assimilation enzymes ATP-S and OAS-TL, in addition to S-containing amino acids content (Cys), which can detoxify As stress (Figure 6A–F). The S-assimilation route precisely begins with the ATP-S enzyme which catalyzes the formation of adenosine phosphosulfate (APS) from ATP and sulfate [83]. The last phase of S-assimilation, which produces Cys from OAS and H_2_S, is catalyzed by OAS-TL. At the 500 mM SA level, SA maximally boosted the activity of S-assimilation enzymes and metabolites, which are essential for providing the defense against As stress. Assimilation of S is the main metabolic process in plants, resulting in the production of amino acids like Cys and methionine, required for the synthesis of a variety of metabolites, including reduced glutathione and metal chelators (NPTs and PCs), all of which are important in the biochemical response to metal/metalloid stress [84]. These S-containing amino acid pools later participate in the demand-driven formation of antioxidants and other defensive biomolecules [36,85]. GSH is well-documented as the first-line defense in response to As stress before the production of PCs [19]. The GSH level can be enhanced with the application of SA, which ultimately increased the PCs content [18,86]. Consequently, the exogenously administration of SA promotes tolerance in plants and results in As detoxification by up-regulating antioxidant enzymes and by including the biosynthesis of heavy metal chelators like PCs and NPTs, which reduced oxidative stress in *B. napus* cultivars.

## 5. Conclusions

Conclusively, the results show that among SA 250 mM and 500 mM, 500 mM more prominently reduced the unfavorable effects of As stress on photosynthesis and growth potential of the plants. The increased SA supply lowered As-uptake and reduced oxidative stress by reducing ROS accumulation in the plants. The rise in proline accumulation with the exogenous supplementation of SA resulted in the alleviation of As toxicity by osmotic adjustment and protection of enzyme structure. Salicylic acid supplementation significantly reduced the harmful effects of As on plant growth, development, and photosynthesis by increasing enzymatic and non-enzymatic antioxidant defense, S-assimilation, and in addition, the concentration of PCs and NPTs in plants. SA application also enhances the production of carbohydrates, which simultaneously reduces the As-induced stress by increasing the functionality of Rubisco and photosynthesis. Since the problem of As increase in agricultural soils is getting worse quickly, it is imperative to find the strategies and procedures to decrease As toxicity in crops. With the use of different biotechnological and genetic methods, the mechanism behind SA-induced As tolerance may be further investigated.

## Data Availability

Data are contained within the article.
